# “Culture Is So Interspersed”: Child-Minders' and Health Workers' Perceptions of Childhood Obesity in South Africa

**DOI:** 10.1155/2017/9629748

**Published:** 2017-03-01

**Authors:** Roger Figueroa, Jaclyn Saltzman, Jessica Jarick Metcalfe, Angela Wiley

**Affiliations:** ^1^University of Illinois at Urbana-Champaign, 905 South Goodwin Ave., 230 Bevier Hall MC-180, Urbana, IL 61801, USA; ^2^University of Illinois at Urbana-Champaign, 904 W. Nevada St., #1014, MC-081, Urbana, IL 61801, USA; ^3^University of Illinois at Urbana-Champaign, 904 W. Nevada St., #2004, MC-081, Urbana, IL 61801, USA

## Abstract

*Introduction.* Forty-one million children globally are overweight or obese, with most rapid rate increases among low- and middle-income nations. Child-minders and health workers play a crucial role in obesity prevention efforts, but their perceptions of childhood obesity in low- and middle-income countries are poorly understood. This study aims to (1) explore child-minders and health workers' perceptions of the causes, consequences, potential strategies, and barriers for childhood obesity prevention and intervention in Cape Town, South Africa and (2) to provisionally test the fit of a socioecological framework to explain these perceptions.* Methods.* Twenty-one interviews were recorded, transcribed, and analyzed through analytic induction.* Results.* Participants identified multilevel factors and contexts, as well as potential consequences and priorities of interest in addressing childhood obesity. An adapted childhood obesity perceptions model was generated, which introduces an overarching cultural dimension embedded across levels of the socioecological framework.* Conclusions.* Culture plays a pivotal role in explaining obesogenic outcomes, and the results of this study demonstrate the need for further research investigating how obesity perceptions are shaped by cultural frames (e.g., social, political, and historical). Understanding the causes, consequences, and potential interventions to address obesity through a cultural lens is critical for promoting health in low- and middle-income nations.

## 1. Introduction

The World Health Organization reports that nearly 41 million children globally are overweight or obese, with the most rapid increases in overweight and obesity rates reported in low- and middle-income nations [1]. As low-income countries shift to participate more in the global economy, obesity and malnutrition emerge as simultaneous health burdens [2]. Low- and middle-income countries such as South Africa have a high rate of childhood obesity across all economic levels and age groups [3], with rural youth affected most by underweight and urban youth affected most by overweight [4]. It is important to note that South Africa has been recently classified as an upper-middle income country but has a significant number of low-income settings within its borders, due in part to the 23.9% unemployment rate [5].

Childhood obesity is a particularly challenging phenomenon because it predicts obesity in adulthood, as well as cardiovascular and metabolic disease, but is resistant to treatment in the majority of cases [6–8]. South Africa has the highest obesity rates in Africa [3, 9, 10]. Rates of obesity and overweight among South African children are increasing at a similar pace as developed countries in prior decades at 13.5% [11], suggesting further research on implementing successful interventions to address this issue is needed.

Given the country's complicated history embedded in its cultural, economic, and political landscapes, it is imperative that researchers and policymakers avoid overgeneralizing Westernized views and data when designing interventions for childhood obesity in South Africa. Specifically, the effects of Apartheid-era policies and current unemployment and homelessness rates [12, 13] may reduce the effectiveness of intervention and prevention programs that were developed in high-income nations with different economic, health, and education infrastructure. It is also important to highlight South Africa's racial and cultural make-up, including Native Africans, European descendants (whites), and coloured (predominantly mixed race descendants from East and Central Africa) as well as Asian descendants [14]. Baseline data on South African perceptions related to childhood obesity are needed to underpin effective intervention in this multifaceted context.

**Figure 1:**
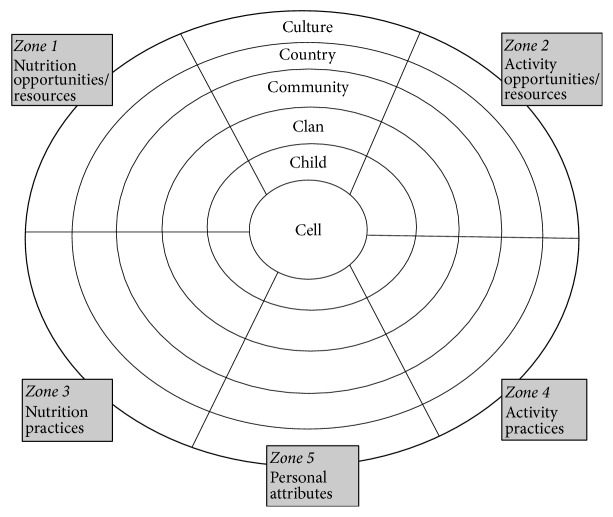
The Six C's Ecological Developmental Model of contributors to overweight and obesity in childhood [15].

Risk for childhood obesity cannot be traced to one specific factor but is rather the result of numerous interacting contributors across levels of influence, as previously proposed in the Six C's Model [15]. Bronfenbrenner's ecological model provides grounding for this transdisciplinary framework that explores risk and protective factors for childhood obesity, at the cellular, child, clan, community, country, and cultural levels (Figure 1). The Six C's Model of childhood obesity presents a framework for considering these contributing factors across concentric levels of influence [15]. Harrison and colleagues summarized the literature on biological (cell), psychological/behavioral (child), social and familial (clan), economic and political (community and country), and cultural predictors of childhood obesity in the United States in order to create the Six C's Model. The Six C's Model, like other socioecological approaches, conceptualizes the individual as embedded within the context of development over time and poses that factors within the concentric contexts are bidirectionally influential [15]. For example, a factor within the community sphere (e.g., school lunch availability and nutritional quality) is affected by the national economy (country sphere) and in turn has the potential to affect family finances (clan), child eating behavior (child), and ultimate adiposity (cell). However, the model's generalizability across cultures has not yet been explored. For the purposes of this study, culture can be defined as a system of shared and learned patterns of thoughts consisting not only of practical information about the world but also of values, attitudes, and beliefs [16].

Culturally rich accounts are important because—depending on a country's historical, political, social, and economic context—fat (or obesity) serves as a cultural indicator of such disparate characteristics as benevolence, greed, wealth, success, power, and failure [17]. Simply put, culture shapes how individuals perceive obesity [18, 19]. These and other interacting contributors across socioecological levels play a crucial role in how individuals may perceive obesity.

It is important to examine* perceptions* about childhood obesity using a theoretical framework as a first step toward addressing multiple factors contributing to childhood obesity. Understanding how members of the public perceive the causes of obesity promotes discussion of relevance to public health practitioners. Specifically, theory-based research on obesity perceptions will allow researchers to identify whether key figures in South African obesity prevention efforts will view obesity according to the relatively well-accepted social constructions of the phenomenon (e.g., obesity as “epidemic,” socially acceptable discrimination of obese individuals) [20]. The job of public health and medical professionals is to provide an avenue to healthful behavior, sensitive to public perceptions of what it means to be obese. However, given that obesity is a health issue complicated by social stigma that varies from culture to culture, it is critical that practitioners are aware of how obesity is perceived. To be obese is not simply to be perceived as being fat, or not thin, or not fitting some societally proscribed standard of beauty. To be obese is to have a ratio of weight for height that is strongly correlated with increased risk for cardiovascular disease, stroke, diabetes, or arthritis in joints [21]. To be obese is to be told to lose weight or suffer the consequences described above [22], but then to be given no viable long-term options for the prescribed weight loss [23]. Thus, culturally informed examination of perceptions of obesity is critical in order for researchers and practitioners to identify promising avenues for prevention and intervention.

Understanding patient perceptions about obesity and its consequences may give providers opportunities to prescribe health behavior change to their patients in a sensitive manner (e.g., taking into account patients readiness to engage in healthy habits for health versus lose weight). Using objective indicators—such as cholesterol levels, blood pressure, or oxygen uptake—to discuss obesity-related health problems may be a more fruitful path to behavior change than focusing on weight loss alone [24]. Importantly, it is clear that shaming patients into weight loss is not an effective method to change nutrition and physical activity behaviors [25]. In order to change health behaviors at the individual and family levels, researchers must first identify how relevant stakeholders perceive obesity in specific communities and cultures, before prevention and intervention efforts can be developed or addressed.

Child-minders (called child care providers in some contexts) and health workers are important components of the community sphere in the Six C's Model, because they provide valuable health information to patients, clients, and community members. Both groups have access to children and their parents and provide support for children's health and well-being. Community-based prevention tools can be effectively implemented in their places of work including schools, hospitals, early childhood education programs, and through the media [3, 26]. A study exploring perceptions about risk factors for obesity among low-income Latinos in Texas found parents, caregivers, and health workers as important players in preventing obesity among children [27]. Health workers are central to the prevention and treatment of childhood obesity; parents, schools, and society in general consistently turn to providers regarding weight-related health [28]. Given this and considering the limited time spent during medical training on nutrition and physical activity [29], clinicians are prime targets for intervention [30].

While child-minders and health workers are important agents in the health ecology of children, little research has tested the fit of novel theories to their perceptions about the causes and consequences of childhood obesity. Even less work has explored this among care providers in low- and middle-income countries. Being able to explore the current perceptions of these professionals using a sound theoretical framework would allow for identification of areas for further intervention and involvement of stakeholders in addressing childhood obesity.

This study uses an existing socioecological framework to explore the current perceptions of childhood obesity among a purposive sample of child-minders and health workers in Cape Town, South Africa. Our first aim (Aim 1) is to qualitatively explore perceived causes and consequences of, as well as the perceived strategies for and barriers to, addressing childhood obesity among health workers and child-minders in Cape Town, South Africa. Our second aim (Aim 2) is to examine whether the perceptions and understandings of childhood obesity among child-minders and health workers can be organized according to a preexisting socioecological model of childhood obesity (Figure 1). Focusing on these objectives using a qualitative approach will support further initiatives in testing the relationship between the theoretical fit of actual and perceived factors associated with childhood obesity in low- and middle-income countries.

## 2. Materials and Methods

Recently, there has been a call for more qualitative work focused on obesity that allows for more detailed and nuanced insights about obesity [31]. More specifically, Perez and Ball [31] have found that, of current publications focused on obesity, qualitative studies only account for approximately 1% of the total studies. This study aims to reduce this gap in the literature by qualitatively examining perceptions of obesity among South Africans.

### 2.1. Research Design

Semistructured in-depth interviews were conducted between January and June 2014 in Cape Town, South Africa. We used an adapted obesity perceptions interview protocol [27] with questions organized by levels and constructs of a transdisciplinary (TD) conceptual framework (Six C's) to explore participants' perceptions of the multiple levels of influence on children's obesity risk (see the following list). In this study, the target population was child-minders and health workers. The research ethics board at the University of Illinois at Urbana-Champaign approved this study (IRB Reference number: 14411).

*Adapted Semistructured Facilitation Protocol for Conducting Interviews [27]*

1. Introductory Remarks
  (1a) Introductions
  (1b) Consent forms
  (1c) Ground rules: timing, reinforcement of no right or wrong answers, confidentiality.
  (1d) Audio-recording initiation (if applicable)
2. Exploration of childhood obesity perceptions
3. In general, what would you say are the main causes to “overweight or obesity” in children?
4. Probing questions:
5. What consequences are you most concern about?
6. How concerned are you about childhood obesity in your community? Why?
7. What roles do the following play in child obesity? If possible, please expand as to why.
  (2a) What is the role of a child or an individual in childhood obesity?
  (2b) What are the roles of family and friends in childhood obesity?
  (2c) What are the roles of parents in childhood obesity?
  (2d) What are the roles of grandparents in childhood obesity?
  (2e) What are the roles of childcare providers (child-minders) in childhood obesity?
  (2f) What are the roles of health Provider in childhood obesity?
  (2g) What is the role of the community (e.g., schools, environment) in child obesity?
  (2h) What is the role of the government in childhood obesity?
  (2i) What is the role of media in childhood obesity?
  (2j) What is the role of culture in childhood obesity?
8. Probing questions:
9. How and why they play a role in child obesity?
10. In your opinion, what would be helpful for the community you work with to prevent childhood obesity?
  (3a) Are there any potential solutions?
  (3b) Are there any intervention strategies to address childhood obesity?
  (3c) Would you name any major barriers to such?
11. Concluding remarks
12. Would participant like to add anything else to this discussion?
  (4a) No further comments conclude the interview.
  (4b) Thank participants very much for being part of this research study!
  (4c) Participants receive incentive valued in $10 as an appreciation token for their time.

### 2.2. Recruitment and Data Collection

Following IRB approval, participants were recruited using a purposive sampling technique (i.e., snowball sampling) [32]. Under the purposive sampling assumptions participants were screened to reflect the following inclusion criteria: (1) willing to do interviews in English, unless they had communicated prior to the face-to-face meeting that they would like to bring a translator on site, (2) over 21 years of age, and (3) currently employed as a health worker or child-minder in the Cape Town area. Project collaborators provided a preestablished list of potential study sites. These sites were contacted using e-mail notifications and face-to-face visits with a recruitment letter describing study criteria. As recruitment of participants who met criteria proceeded, snowball sampling was used to allow participants to identify more participants with similar characteristics (i.e., other known child-minders and health workers) to contribute rich information to this study. This population was of interest to us because they serve as health information outlets [27] and might be key players in the prevention of child obesity [33–37]. Each participant gave written, informed consent prior to participating in the present study. Initially, sample size requirements were informed by Creswell [38, 39], in which is suggested that a sample size between 20 and 30 participants would lead to theoretical saturation. In addition, Francis and colleagues [40] have found that theory-based interview studies including health workers would reach theory-based saturation between interviews 13 and 17. These findings are fairly consistent with Guest and colleagues [41], who reported that the first 12 interviews produced 97% of the main codes in their results.

### 2.3. Participants

There were twenty-one (21) participants in this study. Initially, 13 participants were recruited in January 2014 in Cape Town, South Africa, in the communities of Khayelitsha and Rondebosch. A second round of interviews with 8 new participants took place in May and June 2014 in the community of Heideveld (see Table 1). Interviews took approximately 30 minutes on average. Participants completed a demographic questionnaire, which included questions regarding age, gender, income, years living in South Africa and Cape Town, and language. They self-reported their occupation and education. Individual interviews were conducted by the first author and were audio-recorded (*n* = 16). Participants also had the option to opt out of the audio portion of the interview (*n* = 5) by typing their answers to the interview protocol independently and emailing them to the first author. Participants' remuneration for their time was valued at $10 USD (approximately 150 ZAR). With the exception of one interview, all interviews were conducted in English. A translator was on site to assist in an interview with a child-minder who preferred to communicate in Xhosa.

### 2.4. Data Analysis

Interviews were transcribed verbatim by a professional transcription agency and checked for accuracy by the first author. Using an analytic induction approach, the first author coded the first round of interviews (data collected in January 2014) in the first phase of analysis. Analytic induction is a technique that formulates a set of preliminary hypotheses from analyzing a small number of cases. These are tested and reformulated with the remainder of the available data. It tests a limited number of hypotheses with all available data, in an effort to understand the larger phenomena [45–47]. We formulated preliminary hypotheses for each ecological level of the Six C's Model [15] to test the fit of participants' perceptions about causes and consequences of childhood obesity. We also formulated preliminary hypotheses for each of the open questions about potential solutions and barriers to addressing childhood obesity.

The first author initiated analysis of the data by reading each transcript while identifying general themes (open coding), then coding each transcript line-by-line. Codes were compared and used to form core categories to represent the data. After analyzing the first portion of the data, the research team moved ahead to test the resulting hypotheses. Diagrams were created to illustrate themes and interrelationships between cases, categories, and/or processes [45]. When relevant, “negative cases” that did not fit the emerging hypotheses were analyzed to either revise or add new dimensions to the analysis [47]. Analyses continued until all cases were analyzed. The final interpretation of the data tested a conceptual framework where results were systematically reported in relation to categories derived from data that addressed the research questions [45, 46].

Two coauthors, formally trained in qualitative methodologies, independently coded a subset of cases following the first author's coding system. More specifically, they engaged in initial (line-by-line) and intermediate coding. Then, the research team met to develop a merged coding template. All investigators in the research team were knowledgeable about the Six C's ecological framework and its application and took into account these sensitizing concepts while analyzing the data. Consensus about a final version of this codebook was agreed upon and coding issues, if any, were resolved during the analysis stage via consensus within the research team [48]. During the intermediate coding stage, coauthors independently coded a subset of transcripts; categories that emerged were compared with those identified by the first author. Finally, all investigators triangulated the findings at all stages of data analysis and summarized analyses with a systematic report of the findings and a conceptual framework that represented the most salient concepts in the data from all cases [48].

## 3. Results

### 3.1. Sample Characteristics

In total, five men and sixteen women represented our sample (see Table 2). All men in our sample had occupations in the health field, while child-minders were all women. The average age in our sample was 38.45 years. The majority of participants had lived in South Africa for more than 20 years, with more than half residing in the city of Cape Town. There were no noticeable differences between child-minders and health workers in regard to the length of time they had lived in South Africa. In South Africa, the poverty line as of March 2011 was between 5,316 ZAR ($360 USD) and 7,440 ZAR ($504 USD) annually [49]. This is significantly lower than in the US, where the poverty line is $11,000 a year per person [50], equal to about 150,000 ZAR. Our sample of child-minders reported annual income levels close to the South African poverty line (less than 10,000 ZAR), whereas a majority of health workers reported earning more than 35,000 ZAR annually. In terms of education, child-minders on average had less than a college degree, whereas health workers had at least a college degree. Education varied considerably among participants: most had some high school (*n* = 7), with fewer indicating matriculation (*n* = 2), technical college attendance (*n* = 1), attainment of a university degree (*n* = 6), graduate school attendance (*n* = 3), and unknown (*n* = 2). Participants were all fluent in English with the exception of one, but their primary languages were Swahili (*n* = 1), Afrikaans (*n* = 3), Xhosa (*n* = 5), and English (*n* = 11). Given the inherent socioeconomic differences between child-minders and health workers in this sample, we chose not to further stratify by income or education in our analyses, in order to preserve our focus on participants' perspectives and beliefs as child-minders and health workers, rather than their status.

### 3.2. Aim  1: Evaluating Obesity Perceptions of Child-Minders and Health Workers

#### 3.2.1. Comparisons between Child-Minders and Health Workers

Participants were asked about factors at every level of the Six C's model (Figure 1), and whether they perceived these factors to be associated with childhood obesity outcomes. Findings from the in-depth interviews revealed an array factors associated with obesity: contributing factors to childhood obesity, consequences of childhood obesity, contexts in which contributing factors affect childhood obesity outcomes, and priorities regarding childhood obesity prevention and treatment.

A close examination of the data indicated that there were both differences and similarities between child-minders and health workers in the way they discussed childhood obesity. Child-minders were more likely to mention peer influences on eating behaviors than health workers, likely because they observe children interacting while eating on a daily basis. Health workers were more likely to emphasize nutritional issues common in the South African population (like missing breakfast or nutritional deficits), whereas child-minders focused on more individualized issues contributing to childhood obesity (like overeating and lack of physical activity). Education was mentioned more frequently by health workers, who claimed that many South Africans (children especially) are not well educated about healthy eating and the consequences of childhood obesity. Both groups attended to issues of access and affordability, emphasizing the prevailing effect of socioeconomic situation (SES) on eating and physical activity.

These findings suggest that child-minders were more likely to identify influences at the child (i.e., overeating, physical activity) and community levels (i.e., peer-relationships), whereas health workers were more likely to emphasize influences at the country level (i.e., education, nutritional deficits). Almost all participants noted that healthy foods are usually more expensive and that poorer individuals are more likely to live in dangerous neighborhoods where it is not safe to let children play outside. Strikingly, both child-minders and health workers noted prevailing cultural trends that encouraged obesity among South Africans, for example, the pattern that often obesity is not viewed as problematic but instead is associated with wealth and power.

#### 3.2.2. Combined Participants' Perceptions

Overall, participants were knowledgeable and aware of a range of factors contributing to obesity including biological, behavioral, familial, and environmental influences. Two key themes emerged that were related to biological contributing factors: genetics and malnutrition. In addition, behavioral factors attributed to childhood obesity that emerged in our data were diet and physical activity. In regard to familial factors attributed to childhood obesity, participants gave specific examples of the role of parents, grandparents, and even siblings. Two other key themes emerged regarding environmental factors affecting childhood obesity outcomes: lack of access to healthy foods and limited opportunities for physical activity. With regard to consequences of childhood obesity, participants expressed concern about increased healthcare costs because of high obesity rates, comorbidities, and mortality, as well as increased likelihood of developing noncommunicable diseases such as heart disease, type 2 diabetes, cancer, hypertension, cholesterol, stroke, osteoarthritis, liver, and gallbladder disease.

The interview protocol explored contexts where contributing factors might affect childhood obesity outcomes including schools, community, health facilities, food environments (e.g., fast foods, food deserts), and government. Lastly, priorities for preventing childhood obesity emerged as participants talked about education, physical activity, and safe environments. See the emergent themes and example excerpts in Table 3.

### 3.3. Aim  2: Assessing the Fit of the Six C's Model for Child-Minders and Health Workers' Combined Perceptions of Childhood Obesity Causes and Consequences

**Figure 2:**
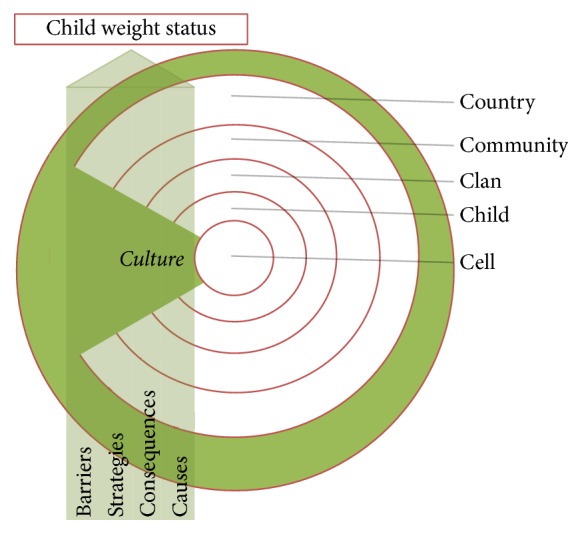
Six C's of childhood obesity perceptions among child-minders and health workers in South Africa.

The multitude of factors that emerged from the in-depth interviews are displayed in Table 4, organized according to the original Six C's theoretical model (Figure 1). Overall, participants identified causal factors and consequences of childhood obesity at each level of the ecology proposed by the Six C's Model, as well as strategies for and barriers to childhood obesity prevention. Participants were able to readily provide examples that support the general fit of the Six C's Model of childhood obesity. However, “culture” was not identified as a concentric sphere but rather was described as a driving influence embedded and infused within each of the other spheres (Figure 2). Therefore, we will propose an adaptation of the original Six C's Model based on our data later in this manuscript. First, we will briefly discuss the causal factors, consequences, strategies, and barriers that child-minders and health workers perceived as influencing childhood obesity, at each level of the original Six C's model (Table 4). Then, we will discuss how culture influences and is infused within each sphere according to our revised model.

At the cell level, hereditary and biological factors, such as genetic predisposition, were perceived as causal factors of childhood obesity. One participant saw cancer as related to obesity, noting, “obesity leads to so many other problems including cancer.” Participants did not discuss strategies for prevention or barriers to such efforts in relation to cell-level characteristics.

At the child level, many factors were discussed as contributing to childhood obesity, including eating habits, food preferences, sedentary habits, screen time, skipping meals, and “happiness.” Consequences at the child level included health risks, negative body image, weight stigma, eating disorders, stress, delayed development, fatigue, low self-esteem, laziness, and suicide. Some general strategies to address obesity at the child level included limiting feeding, monitoring weight status, increasing knowledge, promoting physical activity (playing, dancing, and exercising), weight loss, increasing fruit and vegetable consumption, limiting screen time, and providing snack variety. Barriers at the child level included emotional eating and highly desirable or palatable foods.

At the clan level, force-feeding, grandparents, parenting styles, and “spoiling” children were identified as potential causes of childhood obesity. Interestingly, both happiness and bullying were identified as consequences, in addition to more reactionary consequences, such as strict parent routines and lack of participation in sports. Many strategies were identified for prevention, including concrete ideas such as promoting breastfeeding and home visits by dieticians, and more abstract thoughts such as promoting love and hope. Barriers included family-norms around cleaning one's plate, and lack of quality time.

At the community level, child-minder feeding styles, lack of playground infrastructure, and access to junk foods were identified as causes of childhood obesity. Consequences at this level were more limited and focused on profits for healthcare and scholastic performance in the local communities. In contrast, many concrete strategies that utilized community resources were identified, including gardening programs, health education, and stakeholder's meetings. Finally, barriers to change included poor living conditions, safety, time constraints, attitudes towards obesity, and lack of training.

At the country level, socioeconomic status, media, and food availability were identified as top causes of childhood obesity, whereas consequences included malnutrition and population health outcomes over time. Strategies focused on government-based initiatives (i.e., revising dietary guidelines, providing subsidies, and food stamps), as well as private-sector initiatives (i.e., fortifying foods, increasing healthy food production, and promoting positive media outreach around health behaviors). However, providers identified funding as a prime barrier to government and industry involvement at the national level.

Finally, although our codes are listed under the cultural level in Table 4, it is important to note that the cultural causes, consequences, strategies, and barriers related to childhood obesity were consistently linked to factors at other levels, and participants resisted parsing culture out from other factors. Indeed, when asked how a child's culture plays a role in childhood obesity, one child-minder said: “That's very hard to talk about. Culture is so interspersed. You can't distinguish anymore what was cultural and what was choice. It might not have been the choice of people, just to eat mealie-meal and all that stuff. Part of your culture was to have lots of meat and lots of veggies.”

As in other ecological models, culture is presented in the original Six C's Model as a distal sphere in which other contexts are embedded. In an interesting preliminary finding of this study, the data suggest that cultural influences are present within multiple levels of the child's ecology, interacting with other factors more proximally. We therefore propose that “culture” is a global theme or core category in our adapted model. This core category, “culture is so interspersed,” emerged with the greatest explanatory power in regard to the main phenomenon (i.e., childhood obesity) because (1) it is intertwined with multiple ecological spheres (child, clan, community, and country) (2) and it links central structural categories (i.e., perceived causal factors, consequences, strategies, and barriers) that were identified in the data.

When asked about culture, participants used many words and phrases interchangeably, such as “black culture” or “in South Africa” or “in Cape Town” or “in the Western Cape” or “African culture”. The embedded nature of culture across ecological contexts is apparent in the common references to its prevalent and complex nature, for example, as follows.

### 3.4. Excerpt 1: Jennifer (Pseudonym)

 
1 Interviewer (I): “How does children's
 
2 culture play a role in childhood
 
3 obesity?”
 
4 Jennifer (J): “That's very hard to
 
5 talk about. Culture is so
 
6 interspersed. You can't distinguish
 
7 anymore what was cultural and what was
 
8 choice. It might not have been the
 
9 choice of people, just to eat mealie-
 
10 meal and all that stuff. Part of your
 
11 culture was to have lots of meat and
 
12 lots of veggies. ”
 
13 J: “I really believe that, because
 
14 when I go to [inaudible], to events
 
15 there, it's lots of veggies on my
 
16 plate. The unfortunate thing is, the
 
17 way we prepare the food is not okay.”

### 3.5. Excerpt 2: Matthew (Pseudonym)

 
1 Interviewer (I): “And then lastly, the
 
2 culture… [inaudible] How it plays a
 
3 role?”
 
4 Matthew (M): “…A culture is to eat
 
5 like this. We have a specific kind of
 
6 function, and with that function, we
 
7 have this particular – we have many
 
8 functions, cultural functions, as we
 
9 call it, and those functions are very
 
10 much associated with food. And not
 
11 good food. It's overly – it's not bad
 
12 food, but it's cultural food.”

Overall, participants noted how multiple cultures that are embedded within South African society are implicated in eating behaviors. Participants gave examples about the role of “their” culture and black culture specifically in obesity, often in a positive light. Some discussed how healthy habits (i.e., physical activity) may or may not be afforded by one's culturally influenced environment. For example, one individual noted, “I think the culture can help the children. Our culture, we can go to the mountain to fetch the wood, so I walk every day. It can make you lose weight if you are overweight.” Given the prevalence of this “culture” theme and its interrelationship with other ecological levels, we represent culture in our adapted model as a central influence that intersects with other ecological levels (Figure 2).

## 4. Discussion

In sum, participants articulated the multiple contributors to and consequences of obesity as well as potential strategies for and barriers to obesity prevention within South Africa, according to an ecological framework. Culture seems to play a critical role in shaping these perceptions, but also in addressing childhood obesity in this context. This study contributes much needed qualitative data focused on childhood obesity [31] and assesses multiple levels of influence. The aims of this study were to (a) qualitatively explore the perceived causes and consequences of, as well as the potential strategies for and barriers to, addressing childhood obesity among health workers and child-minders in Cape Town, South Africa, and (b) provisionally test the fit of a socioecological theory (the Six C's Model) to explain child-minders' and health workers' perceptions about childhood obesity in South Africa.

As hypothesized, each level of the Six C's Model was validated in the responses of study respondents. In this sense, our model is generally consistent with other scholars' conceptualization of the socioecological factors that influence childhood obesity [15]. Participants were able to articulate an array of multilevel factors and contexts that contribute to obesity outcomes, as well as potential consequences of childhood obesity, and priorities of interest in addressing childhood obesity. This pattern is consistent with previous research targeting perceptions of obesity in ethnic minorities in the United States [27] in which participants were able to identify multiple obesogenic factors affecting their community.

Socioecological models may be particularly useful for understanding childhood obesity in understudied (and underresourced) populations. These models are also powerful for focusing research on different levels of the framework. For example, Kime [51] addresses the socioecological factors at the family level as related to obesity, whereas Sano and colleagues' work [52] explored socioecological factors associated with food insecurity among Latino immigrants via qualitative methods, focusing on the community sphere. The current study supports the importance of examining the specific constellations of contributors and barriers perceived at each ecological level by specific populations. Such an approach could help tailor childhood obesity interventions for greater efficacy in at-risk populations.

Unlike the Six C's Model, in our data, “culture” emerged as a core category embedded within each of the other spheres instead of being the outermost level of the model. Our adapted model of childhood obesity perceptions introduces a cross-cutting cultural dimension in which participants perceive cultural elements to interact in a variety of ways with other factors at varying levels. Though there were a variety of factors, consequences, strategies, and barriers reported at each level of the Six C's Model, many of these factors were described as being culturally influenced. Participants reported different cultural elements related to childhood obesity, such as the role of South African and black culture on the perception of body weight and body image and behavioral habits such as physical activity and eating habits, as well as noting bidirectional effects between culture and other factors at multiple levels of socioecological influence.

These findings support the notion that obesity might be a “culture-bound syndrome.” Ritenbaugh [53] was one of the first scholars to articulate the centrality of culture in shaping obesity. The emergent core category in our study (“culture is so interspersed”) illustrates the embedded relationship of culture throughout other socioecological factors that affect individual and child weight outcomes. There are various possible constellations and interrelationships between these socioecological factors of interest (e.g., causal factors, consequences, strategies, and barriers) and child weight outcomes. These complex relationships, including potential mediators and moderators, are wound into the cultural milieu. Ritenbaugh's [53] “culture bound perspective” highlights four important features of obesity: it cannot be understood apart from its specific cultural or subcultural context; its etiology summarizes and symbolizes core meanings and behavioral norms; its diagnosis relies on culture-specific technology and ideology; and successful treatment is accomplished by participants embedded within the surrounding culture. Given these key features of childhood obesity, it is not surprising that each aspect was present in our data. Ritenbaugh emphasizes each culture's uniqueness, as well as their particular genetic and environmental pools. If obesity can be understood as a culturally bound syndrome, it is important to acknowledge the implications for obesity related research moving forward.

In an important example, Shugart [17] has argued that culture plays a pivotal role in explaining obesogenic outcomes, but not without having sociopolitical implications. For example, if an individual's culture is the main explanatory factor in predicting their own obesity outcomes, then the onus is on the individual to make behavioral changes contrary to their cultural upbringing. This finding needs to be taken into account carefully to prevent negative consequences when addressing obesity.

In Iran, for instance, researchers have found that the causes of childhood obesity are perceived to relate to macrolevel policy influences, the school environment, sociocultural factors, and family and individual behavioral factors, all acting in combination [54]. A key emergent theme in this study was the pervasive influence of the Iranian government's policies on children's food intake and physical activity, in which the political and sociocultural context does not encourage girls and women to participate in active lifestyles [54]. This example emphasizes the point that each culture is unique and needs to be understood within its own context and circumstances.

One significant shortcoming of previous “culture-bound syndrome” research is that it has focused on cultural/popular media images, body shapes, and women weight standards, with little focus on the public health implications of obesity. This study aims to close that gap, promoting translational obesity-related research that aims to improve the health and well-being of individuals, by showcasing the perceptions of individuals working directly with those affected by childhood obesity. Such an orientation provides an opportunity to address health rather than appearance or normative values [28]. Ultimately, we seek to make a contribution beyond just advancing the field of health promotion, by bridging public health and social justice.

This study adds to the scarce qualitative literature that aims to understanding and addressing childhood obesity. Additionally, our interview protocol for assessing obesity perceptions within ecological contexts might be a tool for those interested in exploring childhood obesity and related issues in diverse communities. It could be used also as a resource for initiating dialogue so that perceived contributing factors are understood within their appropriate context. Finally, it could also aid in assessing the cultural background of an individual who intends to engage in health behavior modifications because culture is crucial in order for these efforts to be effective [55].

### 4.1. Study Limitations

There are various limitations that future studies must address. Firstly, there were notable differences between child-minders and health workers: particularly gender, income, and community characteristics. In future studies, it would be ideal to recruit participants in these groups with similar characteristics. Due to constraints during the data collection process, it was difficult to conduct face-to-face interviews with all participants. To allow us to recruit more participants and gather more data, some participants were permitted to respond to the interview questions via e-mail responses. The data are limited in that there may be some variations in responses across these data collection modalities. Lastly, most participants were low- to middle-income. We do observe some variability in the income levels of participants; however, only one participant was from a high-income community (Rondebosch). Additionally, there were noticeable differences in income between child-minders and health workers. This may limit the generalizability of the results in this study. Future studies may cast a wider net to allow comparative analyses.

### 4.2. Implications

The implications of this research shed light on areas that could aid child-minders and health workers in childhood obesity prevention efforts. Previous research indicates that preventative health strategies that incorporate the views of target participants have improved likelihood of success [56]. Health workers and child-minders are important stakeholders, who are familiar with the culture in which their clients live and have important roles relevant for health promotion. They can provide a range of perspectives that can help to identify priorities and directions in planning culturally relevant childhood obesity interventions [57].

Understanding the causes, consequences of and potential solutions to obesity through these and other individuals' cultural lenses could inform tailored, culturally sensitive, and effective intervention programs promoting child health. Working with these professionals in South Africa would require clear communication that attends to cultural norms regarding weight and health behavior.

Our data indicated that South African care providers do not perceive that genetic factors play a central role in childhood obesity, even though these are critically associated with the phenomena in the Six C's Model. Further exploration of perceptions about these various hereditary traits and genetic predispositions is warranted. According to Swinburn and colleagues [58] “the genetic background loads the gun, but the environment pulls the trigger.” Future work should help us better understand how to educate the public about the nuances of genetics and its role in obesity, including emphasizing that acknowledging the role genetics plays does not diminish the involvement of other socioecological factors in the obesity problem (e.g., health behaviors, environmental factors). This research could aid social scientists in bridging the gap in the public eye between genetic research and these other behavioral, social, and environmental factors associated with obesity.

Most importantly, our findings suggest that the role of culture should be taken into account carefully, since culture is both interspersed and widely varied in South Africa. To address the potential challenges of targeting this and other culturally rich populations, researchers and practitioners need to be sensitive to participants' ethnic identities and culture and avoid altering behavior that is closely attached to cultural beliefs. Rather, it would be ideal to add elements to behavioral interventions that leverage culturally rooted strengths in targeted populations. For example, many families perceive mealtimes as times of joy and connection. While eating healthfully is important, severe food restrictions and prohibitions might well be perceived as punishment and be culturally unacceptable. A potential alternative could be modifying environmental features to coexist with cultural practices and encourage positive reinforcement of health behaviors.

## 5. Conclusion

The findings outlined in this study emphasize the pervasive influence of culture on individual, biological, behavioral, familial, and environmental factors that influence childhood obesity. Future studies and obesity prevention efforts should investigate and attend to the key role that culture plays in shaping individual health behaviors. Culturally tailored approaches with key community stakeholders in multiple sectors may be crucial to develop effective strategies addressing childhood obesity prevention efforts.

## Figures and Tables

**Table 1:** Key characteristics of study sites in Cape Town, South Africa.

	Khayelitsha	Rondebosch	Heideveld
Region	Western Cape, CT	Western Cape, CT	Western Cape, CT

Size of study area	10,000/km^2^	2,300/km^2^	9,600/km^2^

Population of study area	391,749	14,591	17,388

Race/Ethnicity & Language information within region^*∗*^	*Race:* Black African (98.6%), Coloured (0.6%); *Language:* Xhosa (90.5%), English (3.2%)	*Race:* White (62.7%), Black African (16.5%), Coloured (9.6%), Indian/Asian (6.1%);* Language:* English (84.3%), Afrikaans (7.6%), Other (8.1%)	*Race:* Black African (5.3%), Coloured (92.3%); *Language:* Afrikaans (63.1%), English (34.4%)

Number of interviewees	12	1	8

Recruitment Strategy	Purposeful; Snowball Sampling		

^*∗*^These data were not collected among participants. Source: [42–44].

**Table 2:** Frequency of demographic characteristics, (*n* = 21).

Category	Average/frequency
Age^**∗**^	38.45
Gender	
Male	5
Female	16
Occupation	
Health worker	8
Child-minder	13
Years in Cape Town^**∗**^	
5 or less	2
6–10	1
11–15	1
16–20	4
20 or more	12
Years in South Africa^**∗**^	
5 or less	—
6–10	—
11–15	2
16–20	—
20 or more	18
Education^**∗**^	
Some high school	7
Matriculation	2
Technical college	2
University degree	6
Graduate degree	3
Did not respond	2
Income^**∗**^ (in ZAR)	
Less than 10.000	8
10.000 to 15.000	2
25.000 to 30.000	1
30.000 to 35.000	1
35.000 or more	8
Primary Language^**∗**^	
Afrikaans	3
Xhosa	5
English	11
Swahili	1

^**∗**^One participant opted to not complete the demographic questionnaire.

**Table 3:** Thematic content of participants' perceptions.

Themes	Example excerpts
*Perceived biological contributing factors*	“Some of them they are fat because of they were born with a fat family.”
	“Other factors contributing to obesity include genetic factors and high rates of stunting in children who later become obese.”
	“But maybe, assuming there is a gene that causes you to want to be, like, you know. But if that same gene were a cancer gene, then you would make a plan to avoid it.” “But when it's a fat gene, like it could possibly that easy, then you're just like: well, my mother was fat. I'm just gonna [inaudible] weigh more. So it's the same concept. Because once they overeat, there are many other problems. It is going to break you down and it's going to be a lot sooner than cancer was going to.”

*Perceived behavioral contributing factors*	“One is when they are finished to eat the food, there is no exercise. That's what they do, is eat the food, after that they do sleeping, so its going to gain the weight faster because there is no activity.”
	“Of course this sometimes contributes to obesity and also if you don't watch a child, he's going to eat and eat and eat.”
	“One is [fat] when they are finished to eat the food, there is no exercise. That's what they do is eat the food, after that [what] they do [is] sleeping so it's [the child] going to gain the weight faster because there is no activity.”

*Perceived familial contributing factors*	“I think it's the way that parents feed their children. They overfeed them.”
	“If my grandmother's fat, I'm fat, oh well, we're just a fat family.”
	“They come across psychological things where they emotionally eat. Mother are, ‘Oh, you're crying, I'll give you chocolate, I'll give you food.'”

*Perceived environmental contributing factors*	“Obviously the crime and that kind of thing does play a role because all the mother ensures that the child is safe while she's at work, so she encourages the child to stay at home because it's safer in the house than on the outside of the house.”
	“Most of the time in low socioeconomic systems... They only have minimal to feed the children, so it's usually the high carb things that sort of fill them up very quickly... So they just feed them what they can afford and it's usually the high carb things that make you pick up very quick weight.”

*Perceived cultural contributing factors*	“That's very hard to talk about. Culture is so interspersed. You can't distinguish anymore what was cultural and what was choice. It might not have been the choice of people, just to eat mealie-meal and all that stuff. Part of your culture was to have lots of meat and lots of veggies.”
	“We have many different cultures. Here in South Africa, we've got many people, living their own ways. We've got Muslims, as well. I think our culture doesn't care about what the children eat. They like the children to be big, in order for people to see that this child's family, they come from a very well–off family, and they are fed all the time, which is not good. It is really not good.”
	“… I said black culture, like myself, so I was born in an age that a man has to get married to a person heavy, not the slim. (Unclear) And whenever you're sick, your wife can head back to bring to the hospital, and if there is any problem, she can… When you're obese, you got power. People don't think there's power in someone that's slim.”

*Perceived consequences*	“Consequences I am concerned about? ALL of them! Diet and level of physical activity affects every aspect of one's body- so this list could actually be much longer… Coronary heart disease, Type 2 diabetes, cancers (endometrial, breast, and colon), hypertension (high blood pressure), high total cholesterol, stroke, liver and gallbladder disease, and osteoarthritis (a degeneration of cartilage and its underlying bone within a joint).”
	“The children you will see they not like, you know children they will always feeling tired, you will see them tired and the children, they get sick. Most of the time that will cause a heart attack and that will cause as well diabetes because diabetes also is caused by obesity. When you eat not good food you will gain a lot of weight and that will cause diabetes.”

*Perceived contextual contributing factors*	“It also encourages the children to eat more and more, because we've got some of the shops where they sell that goods, and then you try to control the nutrition for a child at home but when she or he is at school they can eat everything that has been sold.”
	“The unfortunate thing is, the way we prepare the food is not ok. Steamed, and cooked and boiled in water. Soup is food for poor people. That is something in colored communities, whereas we know that soup is a great way of getting nutrition. But for the longest time, soup wasn't associated with health. It was associated with poverty.”
	“It's unsafe to play in the park.”

*Perceived priorities regarding childhood obesity prevention/treatment*	“Insufficient attention is given to teaching school children about healthy diets and lifestyle. Not enough physical activity is promoted or engaged in at schools.”
	“In some communities, there aren't even sidewalks, so how can you tell mothers to go for a walk to take the children for a walk? How can you walk to the park when you're likely to be assaulted or raped?”

**Table 4:** Factors affecting childhood obesity by the Six C's spheres.

Codes	Cell^a^	Child	Clan	Community	Country	Culture
Causes	Heredity	(i) Eating habits (ii) Food preferences (iii) Sedentarism (iv) Screen time (v) Skipping meals (vi) “Happiness”	(i) Force feeding (ii) Parenting styles (and Grandparents) (iii) Spoiling children	(i) Child-minders' feeding styles (ii) Lack of play infrastructure (iii) Access to junk food (iv) Lack of role models	(i) Socio economic status (ii) Media advertising (iii) Food availability and affordability (iv) Food packaging (v) Geographic location	(i) Cultural foods (ii) Traditional practices (iii) Food preparation (iv) Political (Historical)

Consequences	Cancer	(i) Gaining weight (ii) Health risks (iii) Negative body image (iv) Weight stigma (v) Eating disorders (vi) Stress (vii) Delayed development (viii) Tiredness (ix) Low self-esteem (x) Laziness (xi) Suicide	(i) Happiness (ii) Strict parental routines (iii) Lack of participation in sports (iv) Bullying	(i) Healthcare sector profit (ii) Scholastic performance	(i) Childhood obesity incidence (ii) Malnutrition (iii) Population outcomes	(i) Protective factor of HIV stigma (ii) Cultural beliefs about weight and diet

Strategies	—	(i) Limiting feeding (ii) Monitoring weight status (iii) Increasing knowledge (iv) Playing, dancing, exercising (v) Weight loss (vi) Increasing F & V consumption (vii) Limiting screen time (viii) Snack variety	(i) Cooking (ii) Workshops (iii) Family mealtimes (iv) Breastfeeding (v) Serving sizes (vi) Love & Hope (vii) Role modeling (viii) Social support (ix) Reward systems (x) Home visits by dietitians	(i) Health counseling (ii) Health education (iii) Community gardening (iii) Building fitness facilities (iv) Meal routines at childcare (v) Healthcare stakeholders' meetings (vi) Support groups	(i) Dietary guidelines (ii) Subsidizing food products/supplements (iii) Fortified food offering (iv) Food stamps (v) Gardening (vi) Built environment (vii) Media-related (viii) Food production (ix) Access to media outlets (x) Collaborations across sectors	(i) Walkability (ii) Access to nature (iii) Traditional dances

Barriers		(i) Emotional eating (ii) Palatable/desirable foods	(i) “Clean your plate” (ii) Family structure (iii) Lack of quality time	(i) Living conditions (ii) Lack of neighborhood safety (iii) Time constraints (iv) Lack of training (v) People's attitudes	(i) Lack of options (ii) Funding (iii) Soil contaminants (iv) Food insecurity	(i) Myths about women/black culture (ii) “Big,” “Fat” as healthy, endearing

^a^There were no questions exploring perceptions about the cell-level contribution to childhood obesity prevention.
